# Supplementary material for: Targeting TREM2 signaling shows limited impact on cerebrovascular calcification
Source: Life Sci Alliance. 2024 Oct 28;8(1):e202402796. doi: 10.26508/lsa.202402796 (PMC11519321; doi:10.26508/lsa.202402796)
Supplement: Supplementary file 6 [file LSA-2024-02796_TableS1.docx]

**Supplementary Table 1. Nucleotide sequence for ssDNA probes recognizing *Spp1*.**

| **Probe ID** | **Sequence** |
| --- | --- |
| spp1_1 | GCAGGACTAACTACGACCATGAGATTGG |
| spp1_2 | CGGTGAAAGTGACTGATTCTGGCAGCTCA |
| spp1_3 | AGGAGAAGCTTTACAGCCTGCACCCAGAT |
| spp1_4 | TGCCTGACCCATCTCAGAAGCAGAATCT |
| spp1_5 | CTGACGAATCTCACCATTCGGATGAGTC |
| spp1_6 | GATGAGACCGTCACTGCTAGTACACAAG |
| spp1_7 | CAGACACTTTCACTCCAATCGTCCCTAC |
| spp1_8 | CGAGGTGATAGCTTGGCTTATGGACTGA |
| spp1_9 | AGATGAGGACCTCACCTCTCACATGAAG |
| spp1_10 | ATGCCCTCTGATCAGGACAACAACGGAAAG |
| spp1_11 | CAGCCATGAGTCAAGTCAGCTGGATGAA |
| spp1_12 | ATTCCAAAGAGAGCCAGGAGAGTGCCGAT |
| spp1_13 | CAGTCGGATGTGATCGATAGTCAAGCAAG |
| spp1_14 | CCAGCCTGGAACATCAGAGCCACAAGTTT |
| spp1_15 | CTTCCTGTACAAGAAATGCAAACGGCCAC |
