# Supplementary material for: Targeting TREM2 signaling shows limited impact on cerebrovascular calcification
Source: Life Sci Alliance. 2024 Oct 28;8(1):e202402796. doi: 10.26508/lsa.202402796 (PMC11519321; doi:10.26508/lsa.202402796)
Supplement: Supplementary file 7 [file LSA-2024-02796_TableS2.docx]

**Supplementary Table 2. Primary antibodies used for immunohistochemistry.**

| **Antibody** | **Company** | **Catalogue No:** | **Dilution** |
| --- | --- | --- | --- |
| Goat anti-Osteopontin | R&D Systems | AF808 | 1:100 |
| Rat anti-CD31 | Histonova | DIA-310 | 1:100 |
| Goat anti-IBA1 | Abcam | Ab5076 | 1:600 |
| Rabbit anti-IBA1 | Wako | 019-19741 | 1:600 |
| Rat anti-CLEC7A | Invivogen | mabg-mdect | 1:100 |
| Rat anti-CD68 | Bio-Rad | FA-11 | 1:100 |
| Rabbit anti-Ki67 | Invitrogen | MA5-14520 | 1:50 |
| Rabbit anti-pDAP12 | Produced in house by Alector | 1:100 | |
| Rabbit anti-pSMAD3 | Abcam | ab52903 | 1:100 |
| Rabbit anti-APOE | Lifespan Biosciences | LS-B6780 | 1:100 |
| Rabbit anti-Cathepsin K | Abcam | ab19027 | 1:100 |
